# Supplementary material for: Predicting Immunogenic Epitopes Variation of Envelope 2 Gene Among Chikungunya Virus Clonal Lineages by an In Silico Approach
Source: Viruses. 2024 Oct 29;16(11):1689. doi: 10.3390/v16111689 (PMC11599094; doi:10.3390/v16111689)
Supplement: Supplementary file 1 [file viruses-16-01689-s001.zip › Table S7.pdf]

**Table S7.** Clustering details pMHC structure of the interaction between HLA-A and variant epitopes.

| | Cluster Name | Cluster Density[a] | Average RMSD | Max RMSD | Number Of Elements |
|---|---|---|---|---|---|
| HLA-A02 / $^{259}$FPLANVTCMV$^{268}$ | cluster_1.pdb | 93.9119 | 1.12872 | 5.19349 | 106 |
| | cluster_2.pdb | 49.1811 | 2.66362 | 9.19263 | 131 |
| | cluster_3.pdb | 48.9682 | 3.63501 | 11.026 | 178 |
| | cluster_4.pdb | 44.2698 | 1.17462 | 3.31676 | 52 |
| | cluster_5.pdb | 31.6185 | 3.95339 | 33.5801 | 125 |
| | cluster_6.pdb | 22.2693 | 2.24524 | 6.03868 | 50 |
| | cluster_7.pdb | 16.5192 | 5.02446 | 9.59331 | 83 |
| | cluster_8.pdb | 11.6922 | 12.9146 | 35.0845 | 151 |
| | cluster_9.pdb | 9.19119 | 7.61599 | 27.5291 | 70 |
| | cluster_10.pdb | 4.96313 | 10.8802 | 31.7025 | 54 |
| HLA-A02 / $^{259}$FPLANVTCRV$^{268}$ | cluster_1.pdb | 71.5931 | 2.86341 | 8.1852 | 205 |
| | cluster_2.pdb | 32.5057 | 3.9993 | 26.8873 | 130 |
| | cluster_3.pdb | 25.4562 | 5.30324 | 15.0779 | 135 |
| | cluster_4.pdb | 17.186 | 5.00407 | 12.2285 | 86 |
| | cluster_5.pdb | 14.9838 | 8.87625 | 23.0381 | 133 |
| | cluster_6.pdb | 13.6402 | 8.94418 | 31.3573 | 122 |
| | cluster_7.pdb | 10.698 | 6.0759 | 12.5296 | 65 |
| | cluster_8.pdb | 10.3237 | 5.03697 | 11.9947 | 52 |
| | cluster_9.pdb | 6.30987 | 6.02231 | 17.1514 | 38 |
| | cluster_10.pdb | 4.15426 | 8.18438 | 17.4127 | 34 |
| HLA-A30 / $^{1}$STKDNFNVYK$^{10}$ | cluster_1.pdb | 44.8808 | 6.48384 | 20.9559 | 291 |
| | cluster_2.pdb | 23.2029 | 6.50781 | 17.9789 | 151 |
| | cluster_3.pdb | 22.0146 | 2.4075 | 5.70415 | 53 |
| | cluster_4.pdb | 21.5733 | 4.12547 | 12.9283 | 89 |
| | cluster_5.pdb | 15.2558 | 5.44056 | 21.7903 | 83 |
| | cluster_6.pdb | 15.1661 | 6.59366 | 17.3731 | 100 |
| | cluster_7.pdb | 14.1989 | 5.77512 | 14.9389 | 82 |
| | cluster_8.pdb | 12.3725 | 7.75912 | 29.7117 | 96 |
| | cluster_9.pdb | 4.45401 | 4.93937 | 10.1646 | 22 |
| | cluster_10.pdb | 4.34347 | 7.59762 | 29.5518 | 33 |
| HLA-A30 / $^{1}$SIKDHFNVYK$^{10}$ | cluster_1.pdb | 147.239 | 0.346376 | 1.03315 | 51 |
| | cluster_2.pdb | 88.4869 | 0.553754 | 2.82357 | 49 |
| | cluster_3.pdb | 39.6854 | 4.71206 | 15.2851 | 187 |
| | cluster_4.pdb | 36.7878 | 3.28913 | 18.773 | 121 |
| | cluster_5.pdb | 35.7202 | 7.95068 | 31.2235 | 284 |

| | | | | | |
|---|---|---|---|---|---|
| | cluster_6.pdb | 23.5917 | 5.38325 | 17.7636 | 127 |
| | cluster_7.pdb | 10.3675 | 8.48809 | 22.817 | 88 |
| | cluster_8.pdb | 5.14898 | 9.51645 | 16.1724 | 49 |
| | cluster_9.pdb | 4.59822 | 6.52427 | 22.7661 | 30 |
| | cluster_10.pdb | 1.40151 | 9.98922 | 15.4195 | 14 |

These clustering model was predicted by CABS-dock (https://biocomp.chem.uw.edu.pl/CABSdock/).

a; Cluster density is N/Average of RMSD between cluster element; where N = number of element of cluster
