# Supplementary material for: Predicting Immunogenic Epitopes Variation of Envelope 2 Gene Among Chikungunya Virus Clonal Lineages by an In Silico Approach
Source: Viruses. 2024 Oct 29;16(11):1689. doi: 10.3390/v16111689 (PMC11599094; doi:10.3390/v16111689)
Supplement: Supplementary file 1 [file viruses-16-01689-s001.zip › Figure S1_revised.pptx]

## Slide 1
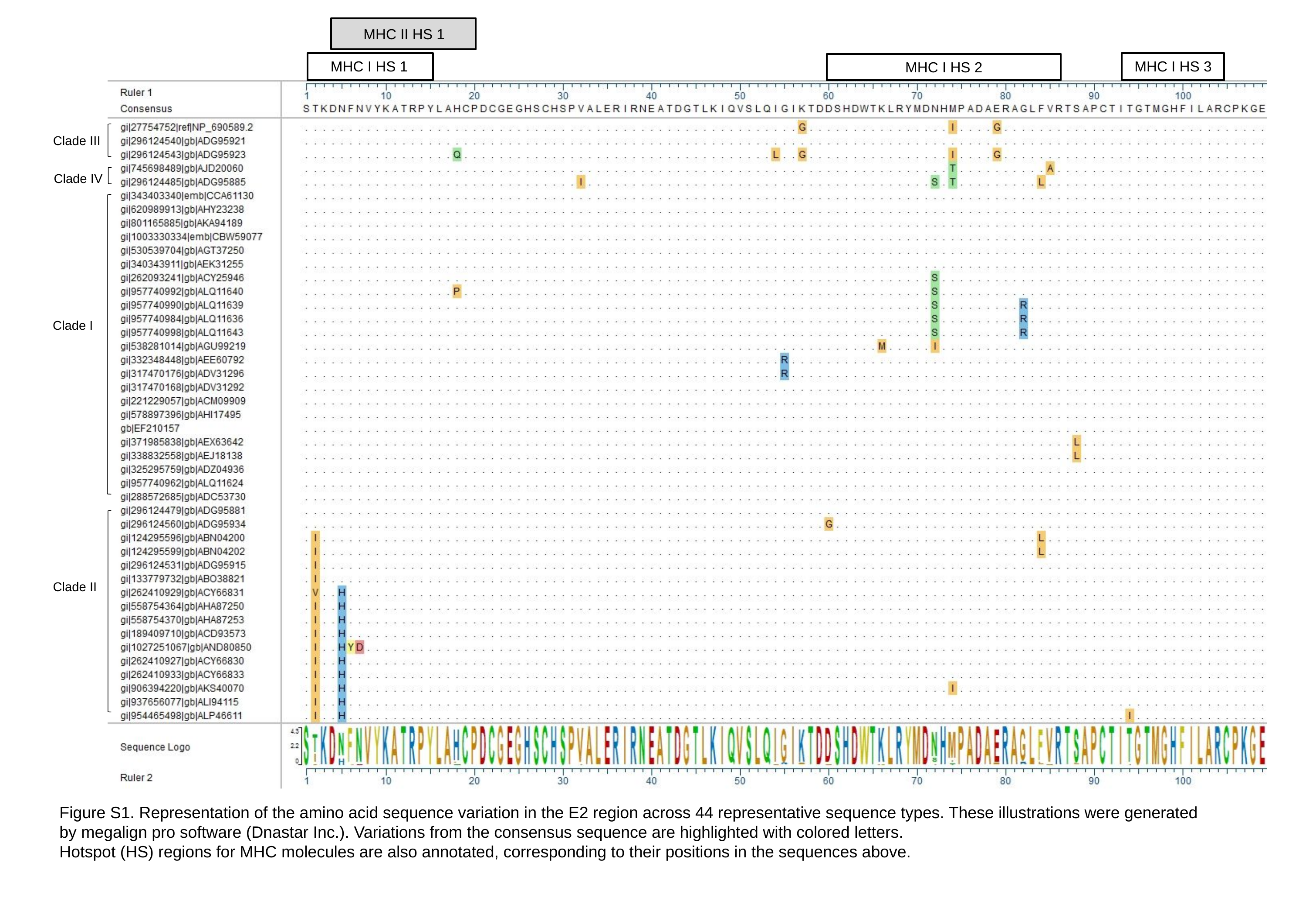

MHC II HS 1
MHC I HS 1
MHC I HS 3
MHC I HS 2
Clade III
Clade IV
Clade I
Clade II
Figure S1. Representation of the amino acid sequence variation in the E2 region across 44 representative sequence types. These illustrations were generated
by megalign pro software (Dnastar Inc.). Variations from the consensus sequence are highlighted with colored letters.
Hotspot (HS) regions for MHC molecules are also annotated, corresponding to their positions in the sequences above.

## Slide 2
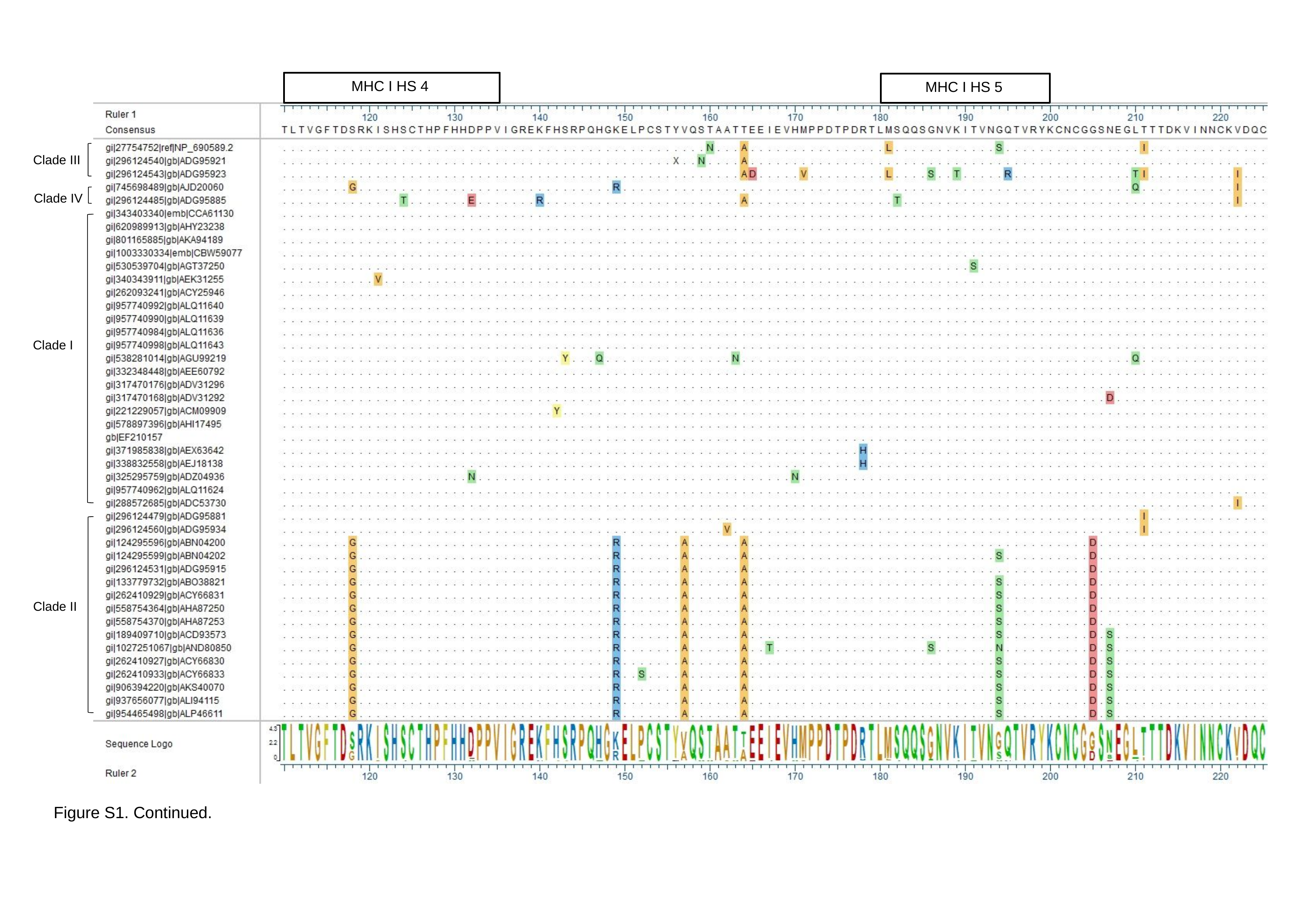

MHC I HS 4
MHC I HS 5
Clade III
Clade IV
Clade I
Clade II
Figure S1. Continued.

## Slide 3
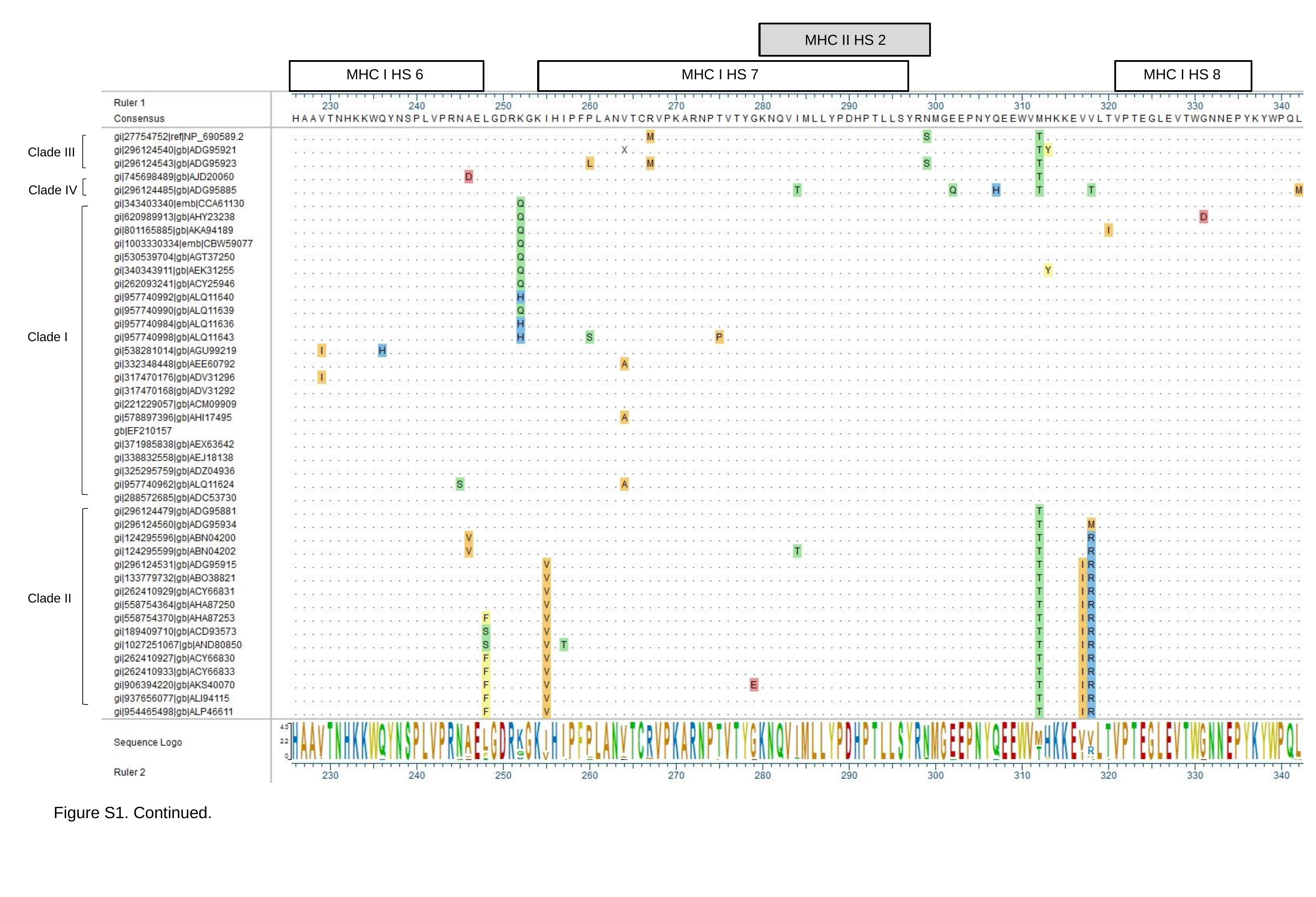

MHC II HS 2
MHC I HS 6
MHC I HS 7
MHC I HS 8
Clade III
Clade IV
Clade I
Clade II
Figure S1. Continued.
