# Supplementary material for: Predicting Immunogenic Epitopes Variation of Envelope 2 Gene Among Chikungunya Virus Clonal Lineages by an In Silico Approach
Source: Viruses. 2024 Oct 29;16(11):1689. doi: 10.3390/v16111689 (PMC11599094; doi:10.3390/v16111689)
Supplement: Supplementary file 1 [file viruses-16-01689-s001.zip › Figure S2_revised.pptx]

## Slide 1
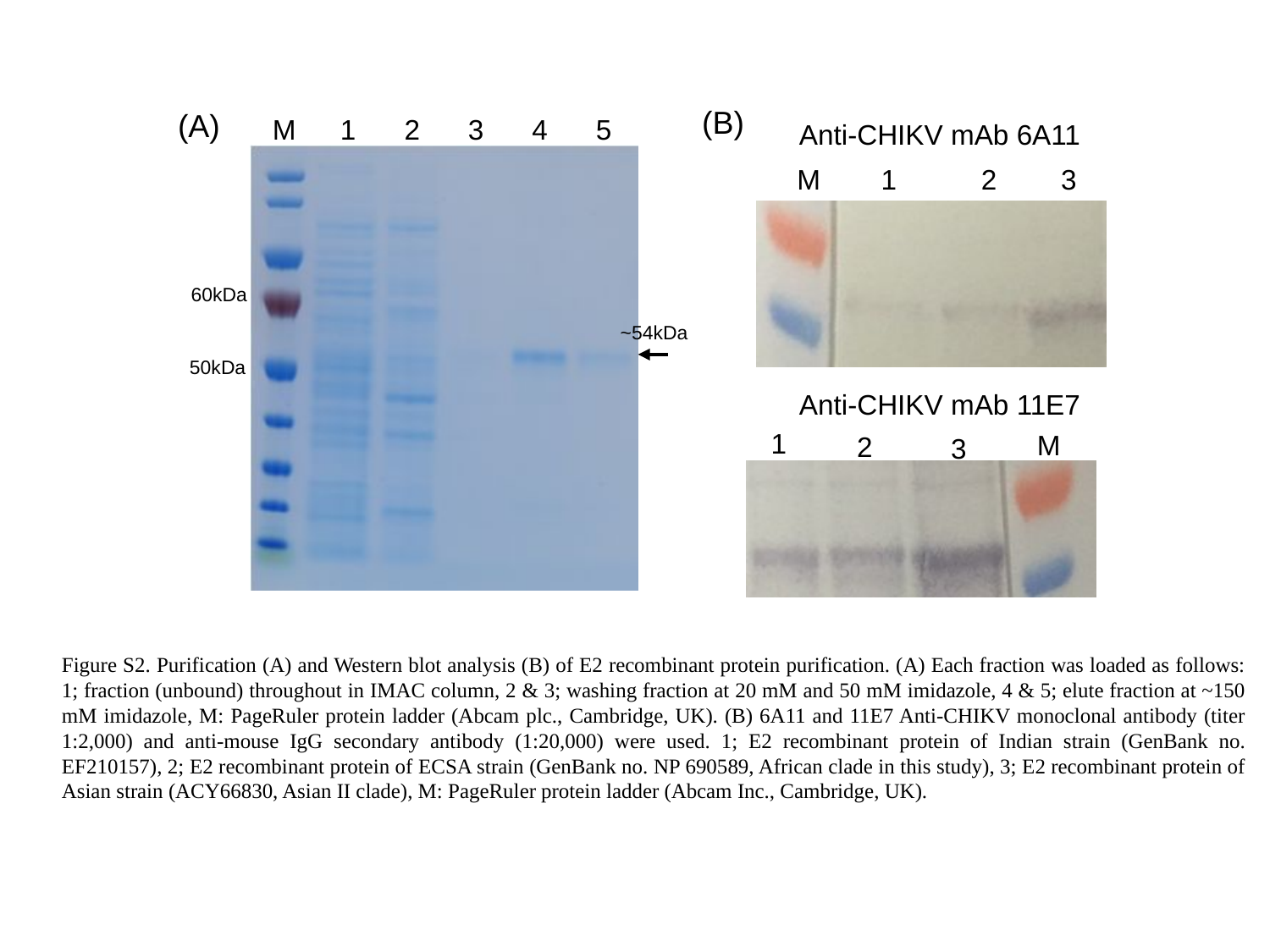

(B)
(A)
M
1
2
3
4
5
Anti-CHIKV mAb 6A11
Anti-CHIKV mAb 11E7
M
1
2
3
60kDa
~54kDa
50kDa
1
2
M
3
Figure S2. Purification (A) and Western blot analysis (B) of E2 recombinant protein purification. (A) Each fraction was loaded as follows: 1; fraction (unbound) throughout in IMAC column, 2 & 3; washing fraction at 20 mM and 50 mM imidazole, 4 & 5; elute fraction at ~150 mM imidazole, M: PageRuler protein ladder (Abcam plc., Cambridge, UK). (B) 6A11 and 11E7 Anti-CHIKV monoclonal antibody (titer 1:2,000) and anti-mouse IgG secondary antibody (1:20,000) were used. 1; E2 recombinant protein of Indian strain (GenBank no. EF210157), 2; E2 recombinant protein of ECSA strain (GenBank no. NP 690589, African clade in this study), 3; E2 recombinant protein of Asian strain (ACY66830, Asian II clade), M: PageRuler protein ladder (Abcam Inc., Cambridge, UK).
