# Supplementary material for: Predicting Immunogenic Epitopes Variation of Envelope 2 Gene Among Chikungunya Virus Clonal Lineages by an In Silico Approach
Source: Viruses. 2024 Oct 29;16(11):1689. doi: 10.3390/v16111689 (PMC11599094; doi:10.3390/v16111689)
Supplement: Supplementary file 1 [file viruses-16-01689-s001.zip › Figure S3_revised.pptx]

## Slide 1
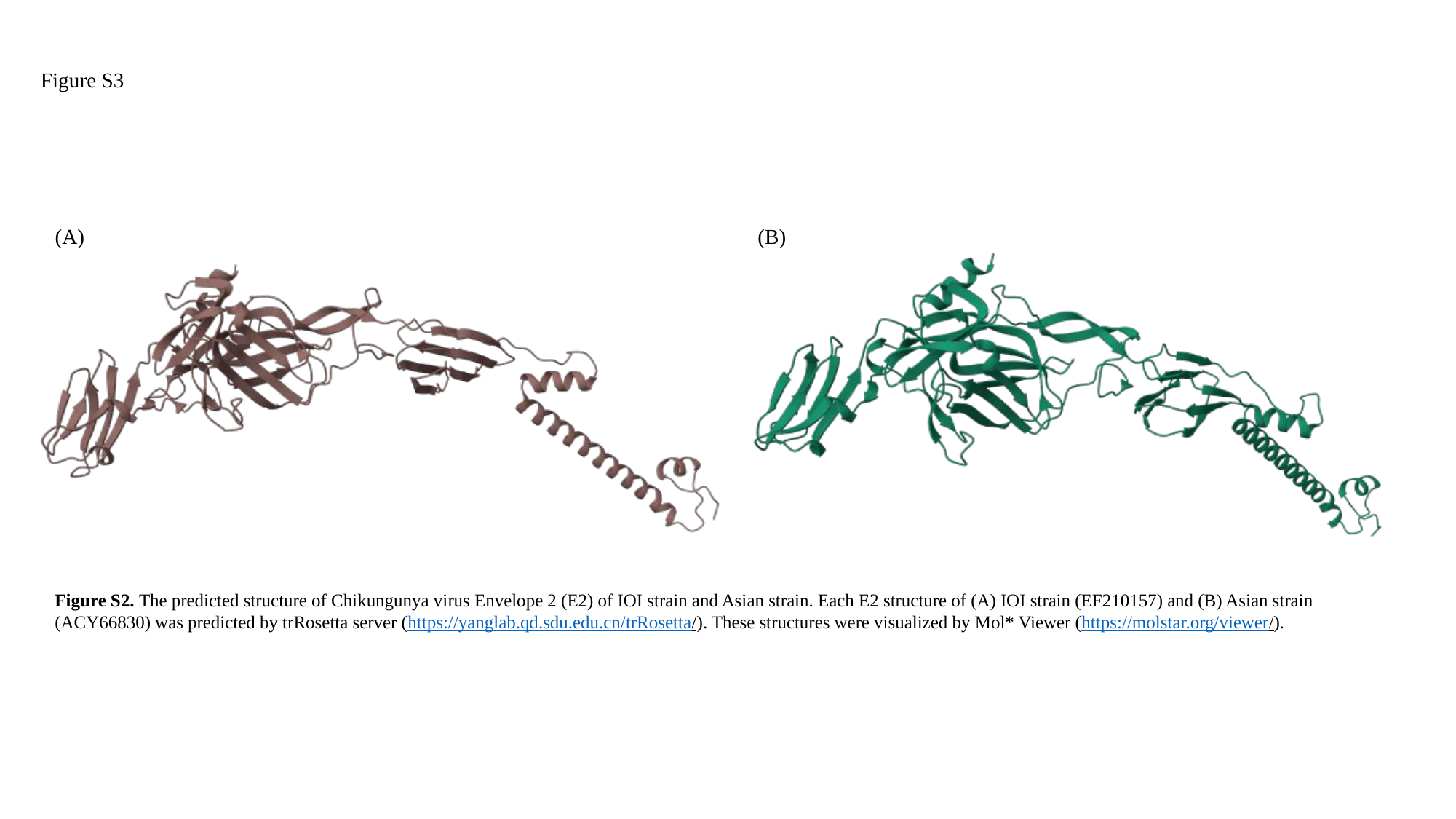

Figure S3
(B)
(A)
Figure S2. The predicted structure of Chikungunya virus Envelope 2 (E2) of IOI strain and Asian strain. Each E2 structure of (A) IOI strain (EF210157) and (B) Asian strain (ACY66830) was predicted by trRosetta server (https://yanglab.qd.sdu.edu.cn/trRosetta/). These structures were visualized by Mol* Viewer (https://molstar.org/viewer/).
