# Supplementary material for: Predicting Immunogenic Epitopes Variation of Envelope 2 Gene Among Chikungunya Virus Clonal Lineages by an In Silico Approach
Source: Viruses. 2024 Oct 29;16(11):1689. doi: 10.3390/v16111689 (PMC11599094; doi:10.3390/v16111689)
Supplement: Supplementary file 1 [file viruses-16-01689-s001.zip › Figure S4_revised.pptx]

## Slide 1
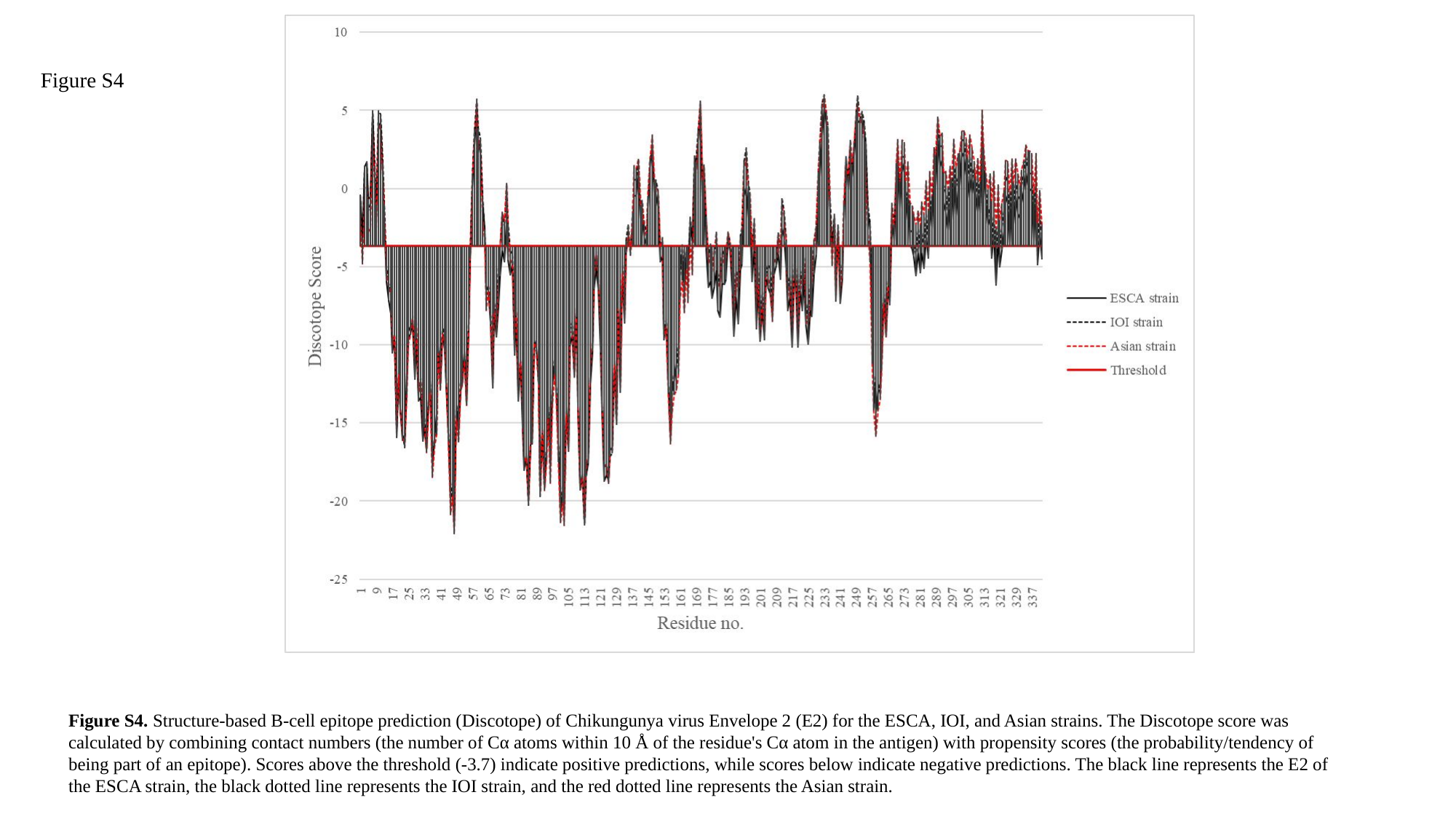

Figure S4
Figure S4. Structure-based B-cell epitope prediction (Discotope) of Chikungunya virus Envelope 2 (E2) for the ESCA, IOI, and Asian strains. The Discotope score was calculated by combining contact numbers (the number of Cα atoms within 10 Å of the residue's Cα atom in the antigen) with propensity scores (the probability/tendency of being part of an epitope). Scores above the threshold (-3.7) indicate positive predictions, while scores below indicate negative predictions. The black line represents the E2 of the ESCA strain, the black dotted line represents the IOI strain, and the red dotted line represents the Asian strain.
