# Supplementary material for: Predicting Immunogenic Epitopes Variation of Envelope 2 Gene Among Chikungunya Virus Clonal Lineages by an In Silico Approach
Source: Viruses. 2024 Oct 29;16(11):1689. doi: 10.3390/v16111689 (PMC11599094; doi:10.3390/v16111689)
Supplement: Supplementary file 1 [file viruses-16-01689-s001.zip › Figure S5_revised.pptx]

## Slide 1
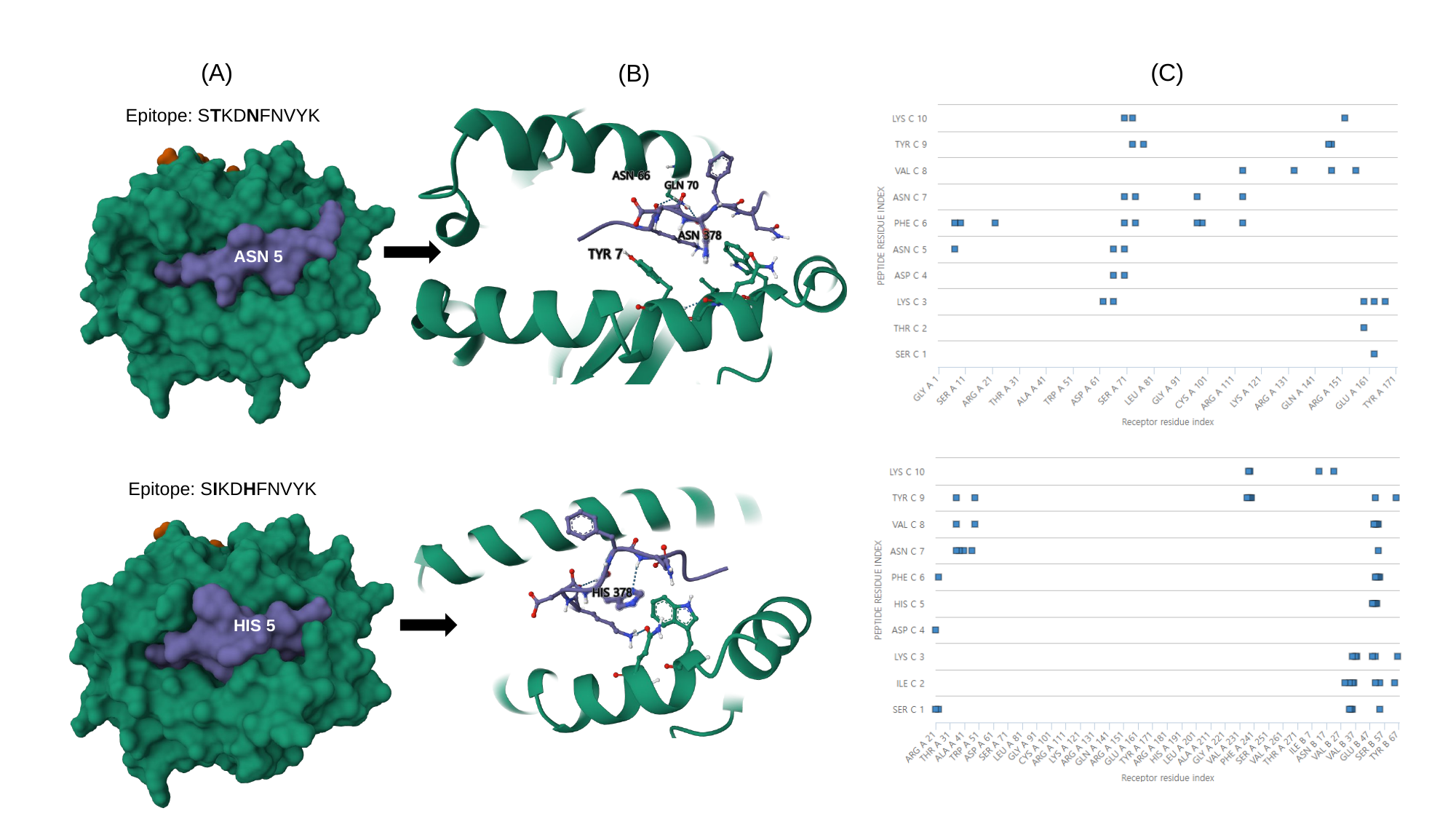

(A)
(C)
(B)
Epitope: STKDNFNVYK
ASN 5
Epitope: SIKDHFNVYK
HIS 5

## Slide 2
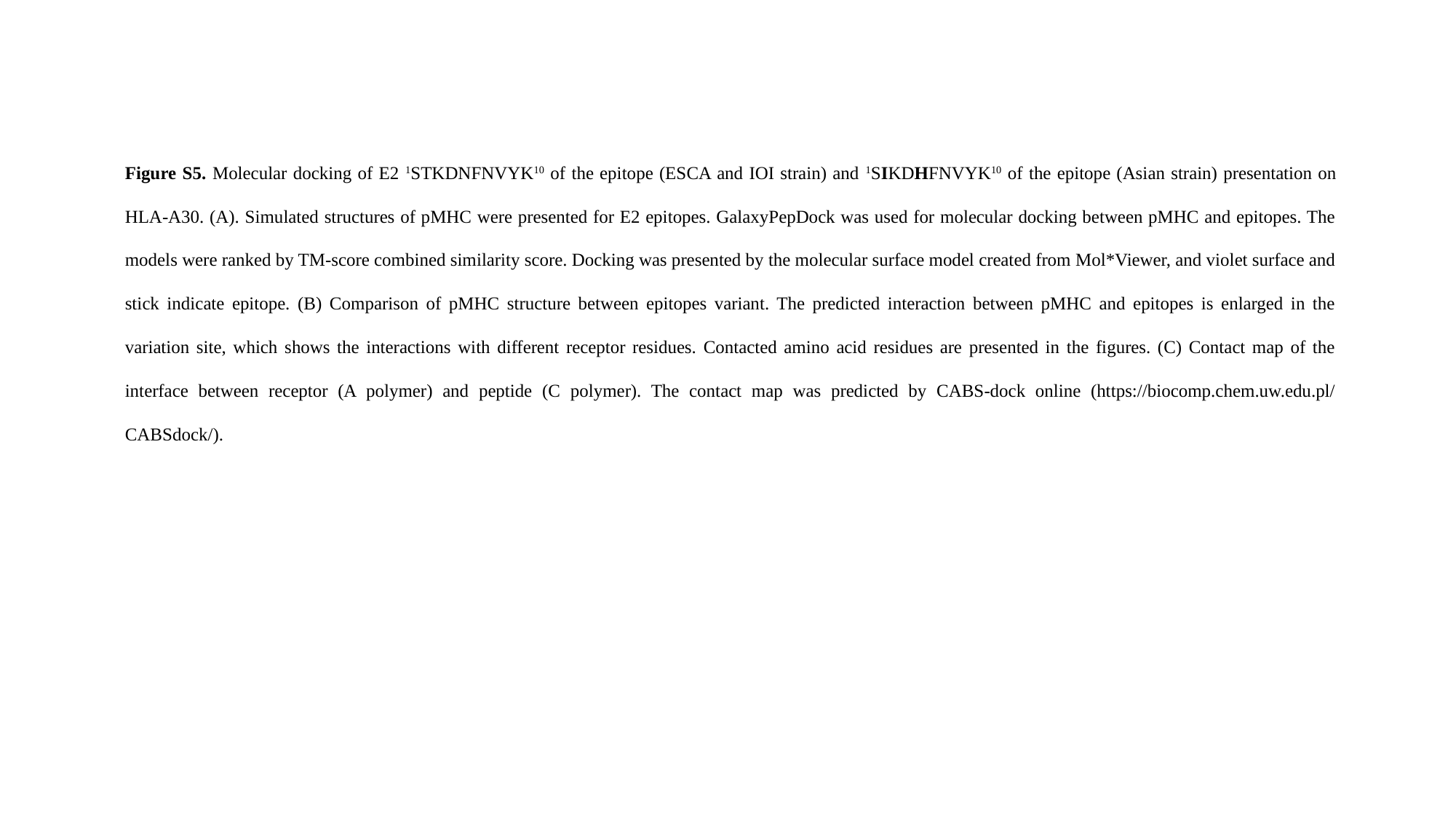

Figure S5. Molecular docking of E2 1STKDNFNVYK10 of the epitope (ESCA and IOI strain) and 1SIKDHFNVYK10 of the epitope (Asian strain) presentation on HLA-A30. (A). Simulated structures of pMHC were presented for E2 epitopes. GalaxyPepDock was used for molecular docking between pMHC and epitopes. The models were ranked by TM-score combined similarity score. Docking was presented by the molecular surface model created from Mol*Viewer, and violet surface and stick indicate epitope. (B) Comparison of pMHC structure between epitopes variant. The predicted interaction between pMHC and epitopes is enlarged in the variation site, which shows the interactions with different receptor residues. Contacted amino acid residues are presented in the figures. (C) Contact map of the interface between receptor (A polymer) and peptide (C polymer). The contact map was predicted by CABS-dock online (https://biocomp.chem.uw.edu.pl/CABSdock/).
