# Supplementary material for: Predicting Immunogenic Epitopes Variation of Envelope 2 Gene Among Chikungunya Virus Clonal Lineages by an In Silico Approach
Source: Viruses. 2024 Oct 29;16(11):1689. doi: 10.3390/v16111689 (PMC11599094; doi:10.3390/v16111689)
Supplement: Supplementary file 1 [file viruses-16-01689-s001.zip › Table S1.pdf]

Table S1. List of B cell linear epitopes of Chikungunya virus E2 protein identified from IEDB assay database (http://www.iedb.org)

IEDB Epitope ID	IEDB Assay ID	Name	Start position of E2	End Position of E2	Method	Qualitative Measure	Heavy chain isotype	PMID
169782	1943008	STKDNFNVYK	1	10	ELISA	Positive	IgG3	22389221
	1943009				inhibition by antigen	Positive	IgG3	
	22731887				microarray	Positive	IgM	38042373
	22731889				microarray	Positive	IgM	
177299	1963301	STKDNFNVYKA	1	11	ELISA	Positive	IgG	23015702
558328	2882343	STKDNFNVYKATRPY + AMID*(Y15)	1	15	ELISA	Positive	IgG	27571254
	2882391				ELISA	Positive	IgG	
558320	2882513	SIKDHFNVYKATRPY + AMID(Y15)	1	15	ELISA	Positive	IgG	27571254
177328	1963302	TKDNFNVYKATRPYLAHC	2	19	ELISA	Positive	IgG	23015702
2248759	22731890	FNVYKATRPY	6	15	microarray	Positive	IgM	38042373
	22731892				microarray	Positive	IgM	
2248700	22731893	ATRPYLAHCP	11	20	microarray	Positive	IgM	38042373
	22731895				microarray	Positive	IgM	
2134495	21242256	TRPYLAHCPDCGEGHSC	12	28	ELISA	Positive	IgG	36016460
2248836	22731896	LAHCPDCGEG	16	25	microarray	Positive	IgM	38042373
					microarray	Positive	IgM	
2248713	22731899	DCGEGHSCHS	21	30	microarray	Positive	IgM	38042373
	22731901				microarray	Positive	IgM	
2248797	22731902	HSCHSPVALE	26	35	microarray	Positive	IgM	38042373
	22731904				microarray	Positive	IgM	
2248912	22731905	PVALERIRNE	31	40	microarray	Positive	IgM	38042373
	22731907				microarray	Positive	IgM	
2248947	22731908	RIRNEATDGT	36	45	microarray	Positive	IgM	38042373
	22731910				microarray	Positive	IgM	
558228	2882403	ATDGTLKIQVSLQIG + AMID(G15)	41	55	ELISA	Positive-Low	IgG	27571254
2248699	22731911	ATDGTLKIQV	41	50	microarray	Positive	IgM	38042373
	22731913				microarray	Positive	IgM	

2248844	22731914	LKIQVSLQIG	46	55	microarray	Positive	IgM	38042373
	22731915				microarray	Positive	IgM	
	22731916				microarray	Positive	IgM	
2134442	21242257	IQVSLQIGIKTDDSHDWT	48	65	ELISA	Positive	IgG	36016460
2248973	22731917	SLQIGIKTDD	51	60	microarray	Positive	IgM	38042373
	22731919				microarray	Positive	IgM	
2248804	22731920	IKTDDSHDWT	56	65	microarray	Positive	IgM	38042373
	22731922		56	65	microarray	Positive	IgM	
2248969	22731923	SHDWTKLRYM	61	70	microarray	Positive	IgM	38042373
	22731925				microarray	Positive	IgM	
2248826	22731926	KLRYMDNHMP	66	75	microarray	Positive	IgM	38042373
	22731928				microarray	Positive	IgM	
2248721	22731929	DNHMPADAER	71	80	microarray	Positive	IgM	38042373
	22731931		71	80	microarray	Positive	IgM	
192864	22731932	ADAERAGLFV	76	85	microarray	Positive	IgM	38042373
	22731934				microarray	Positive	IgM	
2248679	22731935	AGLFVRTSAP	81	90	microarray	Positive	IgM	38042373
	22731937				microarray	Positive	IgM	
2248964	22731938	RTSAPCTITG	86	95	microarray	Positive	IgM	38042373
	22731940				microarray	Positive	IgM	
558231	2882490	CTITGTMGHFILARC + AMID(C15)	91	105	ELISA	Positive	IgG	27571254
	2882491				ELISA	Positive	IgG	
2248711	22731941	CTITGTMGHF	91	100	microarray	Positive	IgM	38042373
	22731942				microarray	Positive	IgM	
	22731943				microarray	Positive	IgM	
2248998	22731944	TMGHFILARC	96	105	microarray	Positive	IgM	38042373
	22731946		96	105	microarray	Positive	IgM	
2248806	22731947	ILARCPKGET	101	110	microarray	Positive	IgM	38042373
	22731949				microarray	Positive	IgM	
2248905	22731950	PKGETLTVGF	106	115	microarray	Positive	IgM	38042373
	22731952				microarray	Positive	IgM	
2248860	22731953	LTVGFTDSRK	111	120	microarray	Positive	IgM	38042373
	22731955		111	120	microarray	Positive	IgM	

558332	2882492<br>2882493	TDSRKISHSCTHPFH + AMID(H15)	116	130	ELISA<br>ELISA	Positive<br>Positive-Low	IgG<br>IgG	27571254
2248990	22731956<br>22731958	TDSRKISHSC	116	125	microarray<br>microarray	Positive<br>Positive	IgM<br>IgM	38042373
2248813	22731959<br>22731961	ISHSCTHPFH	121	130	microarray<br>microarray	Positive<br>Positive	IgM<br>IgM	38042373
193097	22731962<br>22731964	THPFHHDPPV	126	135	microarray<br>microarray	Positive<br>Positive	IgM<br>IgM	38042373
2248793	22731965<br>22731967	HDPPVIGREK	131	140	microarray<br>microarray	Positive<br>Positive	IgM<br>IgM	38042373
558265	2882495	IGREKFHSRPQHGKE + AMID(E15)	136	150	ELISA	Positive-Low	IgG	27571254
2248803	22731968<br>22731970	IGREKFHSRP	136	145	microarray<br>microarray	Positive<br>Positive	IgM<br>IgM	38042373
2248757	22731971<br>22731973	FHSRPQHGKE	141	150	microarray<br>microarray	Positive<br>Positive	IgM<br>IgM	38042373
2248921	22731974<br>22731976	QHGKELPCST	146	155	microarray<br>microarray	Positive<br>Positive	IgM<br>IgM	38042373
2248853	22731977<br>22731979	LPCSTYVQST	151	160	microarray<br>microarray	Positive<br>Positive	IgM<br>IgM	38042373
2249035	22731980<br>22731982	YVQSTAATTE	156	165	microarray<br>microarray	Positive<br>Positive	IgM<br>IgM	38042373
2248672	22731983<br>22731985	AATTEEIEVH	161	170	microarray<br>microarray	Positive<br>Positive	IgM<br>IgM	38042373
192927	22731986	EIEVHMPPDT	166	175	microarray<br>microarray<br>microarray	Positive<br>Positive<br>Positive	IgM<br>IgM<br>IgM	38042373
2248873	22731989<br>22731991	MPPDTPDRTL	171	180	microarray<br>microarray	Positive<br>Positive	IgM<br>IgM	38042373
2248902	22731992<br>22731994	PDRTLMSQQS	176	185	microarray<br>microarray	Positive<br>Positive	IgM<br>IgM	38042373
2248875	22731995	MSQQSGNVKI	181	190	microarray	Positive	IgM	38042373

	22731997				microarray	Positive	IgM	
558257	2882496	GNVKITVNGQTVRYK + AMID(K15)	186	200	ELISA	Positive-Low	IgG	27571254
	2882497				ELISA	Positive	IgG	
2248780	22731998	GNVKITVNGQ	186	195	microarray	Positive	IgM	38042373
	22732000				microarray	Positive	IgM	
2249007	22732001	TVNGQTVRYK	191	200	microarray	Positive	IgM	38042373
	22732003				microarray	Positive	IgM	
2249008	22732004	TVRYKCNCGG	196	205	microarray	Positive	IgM	38042373
	22732005				microarray	Positive	IgM	
	22732006				microarray	Positive	IgM	
2248707	22732007	CNCGGSNEGL	201	210	microarray	Positive	IgM	38042373
	22732008				microarray	Positive	IgM	
	22732009				microarray	Positive	IgM	
2248974	22732010	SNEGLTTTDK	206	215	microarray	Positive	IgM	38042373
	22732012				microarray	Positive	IgM	
2249005	22732013	TTTDKVINNC	211	220	microarray	Positive	IgM	38042373
	22732015				microarray	Positive	IgM	
558343	2882499	VINNCKVDQCHAAVT + AMID(T15)	216	230	ELISA	Positive-Low	IgG	27571254
2249012	22732017	VINNCKVDQC	216	225	microarray	Positive	IgM	38042373
2248831	22732019	KVDQCHAAVT	221	230	microarray	Positive	IgM	38042373
	22732020				microarray	Positive	IgM	
	22732021				microarray	Positive	IgM	
2134434	21242258	HAAVTNHKKWQYNSPLVP	226	243	ELISA	Positive	IgG	36016460
2248792	22732022	HAAVTNHKKW	226	235	microarray	Positive	IgM	38042373
	22732024				microarray	Positive	IgM	
558298	2882500	NHKKWQYNSPLVPRN + AMID(N15)	231	245	ELISA	Positive	IgG	27571254
	2882501				ELISA	Positive	IgG	
2248882	22732025	NHKKWQYNSP	231	240	microarray	Positive	IgM	38042373
	22732027				microarray	Positive	IgM	
2248938	22732028	QYNSPLVPRN	236	245	microarray	Positive	IgM	38042373
	22732030				microarray	Positive	IgM	

2248863	22732031	LVPRNAELGD	241	250	microarray	Positive	IgM	38042373
	22732033				microarray	Positive	IgM	
177356	1963299	VPRNAELGDRKGKIHIPF	242	259	ELISA	Positive-Low	IgG	23015702
	1963300				ELISA	Positive-High	IgG	
2248675	22732034	AELGDRKGKI	246	255	microarray	Positive	IgM	38042373
	22732036				microarray	Positive	IgM	
2248949	22732037	RKGKIHIPFP	251	260	microarray	Positive	IgM	38042373
	22732039				microarray	Positive	IgM	
558260	2882502	HIPFPLANVTCRVPK + AMID(K15)	256	270	ELISA	Positive	IgG	27571254
					ELISA	Positive	IgG	
2248794	22732040	HIPFPLANVT	256	265	microarray	Positive	IgM	38042373
	22732042				microarray	Positive	IgM	
2248838	22732043	LANVTCRVPK	261	270	microarray	Positive	IgM	38042373
	22732045				microarray	Positive	IgM	
2248709	22732046	CRVPKARNPT	266	275	microarray	Positive	IgM	38042373
	22732048				microarray	Positive	IgM	
2248697	22732049	ARNPTVTYGK	271	280	microarray	Positive	IgM	38042373
	22732051				microarray	Positive	IgM	
558348	2882504	VTYGKNQVIMLLYPD + AMID(D15)	276	290	ELISA	Positive	IgG	27571254
	2882505				ELISA	Positive	IgG	
2249020	22732052	VTYGKNQVIM	276	285	microarray	Positive	IgM	38042373
	22732054				microarray	Positive	IgM	
558300	2882466	NQVIMLLYPDHPTLL + AMID(L15)	281	295	ELISA	Positive	IgG	27571254
	2882467				ELISA	Positive	IgG	
2248893	22732055	NQVIMLLYPD	281	290	microarray	Positive	IgM	38042373
	22732057				microarray	Positive	IgM	
2248849	22732058	LLYPDHPTLL	286	295	microarray	Positive	IgM	38042373
	22732060				microarray	Positive	IgM	
2248795	22732061	HPTLLSYRNM	291	300	microarray	Positive	IgM	38042373
	22732063				microarray	Positive	IgM	
2248986	22732064	SYRNMGEEPN	296	305	microarray	Positive	IgM	38042373

	22732066				microarray	Positive	IgM	
192952	22732067	GEEPNYQEEW	301	310	microarray	Positive	IgM	38042373
	22732069				microarray	Positive	IgM	
2249032	22732070	YQEEWVMHKK	306	315	microarray	Positive	IgM	38042373
	22732072				microarray	Positive	IgM	
2249017	22732073	VMHKKEVVLT	311	320	microarray	Positive	IgM	38042373
	22732075				microarray	Positive	IgM	
2248751	22732076	EVVLTVPTEG	316	325	microarray	Positive	IgM	38042373
	22732078				microarray	Positive	IgM	
193118	22732079	VPTEGLEVTW	321	330	microarray	Positive	IgM	38042373
	22732080				microarray	Positive	IgM	
	22732081				microarray	Positive	IgM	
558285	2882506	LEVTWGNNEPYKYWP + AMID(P15)	326	340	ELISA	Positive-Low	IgG	27571254
	2882507				ELISA	Positive-Low	IgG	
2248839	22732082	LEVTWGNNEP	326	335	microarray	Positive	IgM	38042373
	22732084				microarray	Positive	IgM	
192960	22732085	GNNEPYKYWP	331	340	microarray	Positive	IgM	38042373
	22732087				microarray	Positive	IgM	
2248888	22732088	NNEPYKYWPQ	332	341	microarray	Positive	IgM	38042373
	22732090				microarray	Positive	IgM	
558258	2882508	GTAHGHPHEIILYYY + AMID(Y15)	346	360	ELISA	Positive	IgG	27571254
	2882509				ELISA	Positive	IgG	
558261	2882486	HPHEIILYYYELYPT + AMID(T15)	351	365	ELISA	Positive	IgG	27571254
	2882487				ELISA	Positive	IgG	

* Asterisk indicates amidation.
