# Supplementary material for: Predicting Immunogenic Epitopes Variation of Envelope 2 Gene Among Chikungunya Virus Clonal Lineages by an In Silico Approach
Source: Viruses. 2024 Oct 29;16(11):1689. doi: 10.3390/v16111689 (PMC11599094; doi:10.3390/v16111689)
Supplement: Supplementary file 1 [file viruses-16-01689-s001.zip › Table S2.pdf]

Table S2. List of B cell discontinuous epitopes of Chikungunya virus E2 protein identified from IEDB assay database (http://www.iedb.org)

IEDB Epitope ID	IEDB Assay ID	Name	Process Type	E2 position	Response measured	Qualitative Measure	PMID
167045	1938198	V216	Occurrence of infectious disease	216	neutralization	Positive	22144891
	1938199				qualitative binding	Positive	
	1938910				qualitative binding	Positive	
189936	2004510	A487	Occurrence of infectious disease	162	neutralization	Positive	24069479
	2004511				neutralization	Positive	
	2004513				dissociation constant KD	Positive	
	2004514				on rate	Positive	
	2004515				off rate	Positive	
	2004516				qualitative binding	Positive	
	2004517				protection from pathogen challenge after adoptive transfer	Positive	
	2004518				survival after pathogen challenge after adoptive transfer	Positive	
	2004519				qualitative binding	Positive	
189937	2004520	Y394, F409, V438, G439, T441, D442	Occurrence of infectious disease	69, 84,113,114,116,117	neutralization	Positive-Low	24069479
	2004521				neutralization	Positive-Low	
	2004526				qualitative binding	Positive	
	2004529				qualitative binding	Positive	
2224412	22508253	E533, G534, L535, P583	Prophylactic vaccination	208, 209, 210, 258	qualitative binding	Positive	37196061
	22508254				qualitative binding	Positive	

	22508255				qualitative binding	Positive	
	22508256				neutralization	Positive	
	22508257				neutralization	Positive	
	22508258				qualitative binding	Positive	
	22508259				survival from challenge after adoptive transfer	Positive	
	22508265				qualitative binding	Positive-Intermediate	
	22508269				qualitative binding	Positive	
	22508270				decreased disease after adoptive transfer	Positive	
	22508271				pathogen burden after challenge after adoptive transfer	Positive	
	22508457				qualitative binding	Positive	37196061
2224413	22508293	G407, L408	Prophylactic vaccination	82, 83	qualitative binding	Positive	
	22508294				qualitative binding	Positive	
	22508295				qualitative binding	Positive	
	22508296				neutralization	Positive	
	22508297				neutralization	Positive	
	22508298				qualitative binding	Positive	
	22508299				survival from challenge after adoptive transfer	Positive	
	22508302				neutralization	Positive	
	22508305				qualitative binding	Positive	

	22508307				qualitative binding	Positive-Low	
	22508309				qualitative binding	Positive	
	22508447				qualitative binding	Positive-Low	
2224414	22508333	M496, D499, T521, R523, G534, L535, K540, T555, N556, L566, K577, P583	Prophylactic vaccination	171, 174, 196, 198, 209, 210, 215, 230, 231, 232, 233, 258	qualitative binding	Positive	37196061
	22508334				qualitative binding	Positive	
	22508335				qualitative binding	Positive	
	22508336				neutralization	Positive	
	22508337				neutralization	Positive	
	22508338				qualitative binding	Positive	
	22508339				survival from challenge after adoptive transfer	Positive	
	22508342				neutralization	Positive	
	22508345				qualitative binding	Positive-Intermediate	
	22508349				qualitative binding	Positive-Intermediate	
	22508350				decreased disease after adoptive transfer	Positive	
	22508351				pathogen burden after challenge after adoptive transfer	Positive	
2224415	22508353	M496, T521, E533, L535, K540, N556, L566, K577, P583	Prophylactic vaccination	171, 196, 208, 215, 231, 232, 233, 258	qualitative binding	Positive	37196061
	22508354				qualitative binding	Positive	
	22508355				qualitative binding	Positive	
	22508356				neutralization	Positive	
	22508357				neutralization	Positive	

	22508358					qualitative binding	Positive	
	22508359					survival from challenge after adoptive transfer	Positive	
	22508362					neutralization	Positive	
	22508365					qualitative binding	Positive-Intermediate	
	22508369					qualitative binding	Positive-Intermediate	
	22508427					neutralization	Positive-Intermediate	
2224416	22508059	N518, T538, K540, N543	G519, D539, V541,	Prophylactic vaccination	193, 194 ,213, 214, 215, 216, 218	3D structure	Positive	37196061
	22508065					qualitative binding	Positive	
	22508066					qualitative binding	Positive	
	22508067					qualitative binding	Positive	
	22508068					neutralization	Positive	
	22508069					neutralization	Positive	
	22508070					qualitative binding	Positive	
	22508071					survival from challenge after adoptive transfer	Positive	
	22508072					decreased disease after adoptive transfer	Positive	
	22508074					neutralization	Positive	
	22508075					neutralization	Positive-Low	
	22508076					neutralization	Positive-Low	
	22508077					qualitative binding	Positive	
	22508078					qualitative binding	Positive-Low	
	22508079					qualitative binding	Positive-Intermediate	

	22508080				qualitative binding	Positive-Low	
	22508087				pathogen burden after challenge after adoptive transfer	Positive	
	22508120				qualitative binding	Positive	
	22508121				decreased disease after adoptive transfer	Positive	
	22508122				pathogen burden after challenge after adoptive transfer	Positive	
	22508211				pathogen burden after challenge after adoptive transfer	Positive	
	22508456				qualitative binding	Positive	
2224418	22508123	Q509, S510, N512, K514, D539, V541, N543, N544, K546	Prophylactic vaccination	184, 185, 187, 189, 214, 216, 218, 219, 221	3D structure	Positive	37196061
	22508124				qualitative binding	Positive	
	22508125				qualitative binding	Positive	
	22508126				qualitative binding	Positive	
	22508127				neutralization	Positive	
	22508128				neutralization	Positive	
	22508129				qualitative binding	Positive	
	22508130				survival from challenge after adoptive transfer	Positive	
	22508131				decreased disease after adoptive transfer	Positive	

	22508132				neutralization	Positive-Low	
	22508133				neutralization	Positive	
	22508134				neutralization	Positive-Low	
	22508135				qualitative binding	Positive-Intermediate	
	22508136				qualitative binding	Positive	
	22508137				qualitative binding	Positive-Low	
	22508138				qualitative binding	Positive-Intermediate	
	22508139				pathogen burden after challenge after adoptive transfer	Positive	
	22508140				qualitative binding	Positive	
	22508141				decreased disease after adoptive transfer	Positive	
	22508142				pathogen burden after challenge after adoptive transfer	Positive	
	22508208				neutralization	Positive-Low	
	22508209				qualitative binding	Positive-Intermediate	
	22508210				pathogen burden after challenge after adoptive transfer	Positive	
2224419	22508213	R444	Prophylactic vaccination	219	qualitative binding	Positive	37196061
	22508214				qualitative binding	Positive	
	22508215				qualitative binding	Positive	
	22508216				neutralization	Positive	

	22508217				neutralization	Positive	
	22508218				qualitative binding	Positive	
	22508219				survival from challenge after adoptive transfer	Positive	
	22508222				neutralization	Positive	
	22508225				qualitative binding	Positive	
	22508229				qualitative binding	Positive	
	22508230				decreased disease after adoptive transfer	Positive	
	22508231				pathogen burden after challenge after adoptive transfer	Positive	
	22508455				qualitative binding	Positive	37196061
2224420	22508313	R523, G534, L535, P583	Prophylactic vaccination	198, 209, 210, 258	qualitative binding	Positive	
	22508314				qualitative binding	Positive	
	22508315				qualitative binding	Positive	
	22508316				neutralization	Positive	
	22508317				neutralization	Positive	
	22508318				qualitative binding	Positive	
	22508319				survival from challenge after adoptive transfer	Positive	
	22508322				neutralization	Positive	
	22508324				qualitative binding	Positive	
	22508325				qualitative binding	Positive	

	22508327				qualitative binding	Positive-Intermediate	
	22508329				qualitative binding	Positive	
	22508330				decreased disease after adoptive transfer	Positive	
	22508331				pathogen burden after challenge after adoptive transfer	Positive	
2224421	22508273	R523, G534, L535, T555	Prophylactic vaccination	198, 209, 210, 230	qualitative binding	Positive	37196061
	22508274				qualitative binding	Positive	
	22508275				qualitative binding	Positive	
	22508276				neutralization	Positive	
	22508277				neutralization	Positive	
	22508278				qualitative binding	Positive	
	22508279				survival from challenge after adoptive transfer	Positive	
	22508282				neutralization	Positive	
	22508285				qualitative binding	Positive-Intermediate	
	22508287				qualitative binding	Positive-Intermediate	
	22508289				qualitative binding	Positive	
	22508290				decreased disease after adoptive transfer	Positive	
	22508291				pathogen burden after challenge after adoptive transfer	Positive	

	22508426				neutralization	Positive-Intermediate	
	22508458				qualitative binding	Positive	
2224422	22508373	T521, R523, L535, V554, N556, H557, P583	Prophylactic vaccination	196, 198, 210, 229, 231, 232, 258	qualitative binding	Positive	37196061
	22508374				qualitative binding	Positive	
	22508375				qualitative binding	Positive	
	22508376				neutralization	Positive	
	22508377				neutralization	Positive	
	22508378				qualitative binding	Positive	
	22508379				survival from challenge after adoptive transfer	Positive	
	22508389				qualitative binding	Positive-Intermediate	
	22508428				neutralization	Positive-Intermediate	
2224423	22508233	Y334, W389	Prophylactic vaccination	9, 64	qualitative binding	Positive	37196061
	22508234				qualitative binding	Positive	
	22508235				qualitative binding	Positive	
	22508236				neutralization	Positive	
	22508237				neutralization	Positive	
	22508238				qualitative binding	Positive	
	22508239				survival from challenge after adoptive transfer	Positive	
	22508245				qualitative binding	Positive-Low	
	22508249				qualitative binding	Positive	

	22508250				decreased disease after adoptive transfer	Positive	
	22508251				pathogen burden after challenge after adoptive transfer	Positive	
236399	2095960	H398, I399, H424, M496, Q520, T521, Y524, C526, G534, T537, N556, K558	Occurrence of infectious disease	73, 74, 99, 171, 195, 196, 199, 201, 209, 212, 231, 233	qualitative binding	Positive	25275138
	2373552				qualitative binding	Positive	
	2373572				neutralization	Positive-Intermediate	
	2373579				neutralization	Positive	
	2373581				dissociation constant KD	Positive	
	2373670				off rate	Positive	
	2373671				on rate	Positive	
236400	2373554	A487	Occurrence of infectious disease	162	qualitative binding	Positive	25275138
	2373564				qualitative binding	Positive	25275138
	2373574				neutralization	Positive-High	25275138
	2373577				neutralization	Positive-High	25275138
	2373592				dissociation constant KD	Positive	25275138
	2373680				survival after pathogen challenge after adoptive transfer	Positive	25275138
236401	2373551	E349, G380, W389, K391, R405, I446	Occurrence of infectious disease	24, 55, 64, 66, 80, 121	qualitative binding	Positive	25275138
	2373563				qualitative binding	Positive	
	2373571				neutralization	Positive-High	
	2373576				neutralization	Positive-High	
	2373580				dissociation constant KD	Positive	

	2373668				off rate	Positive	
	2373669				on rate	Positive	
	2373679				survival after pathogen challenge after adoptive transfer	Positive	
	2373681				decreased disease after adoptive transfer	Positive	
420680	2095959	E349, G380, W389, K391, R405, I446	Occurrence of infectious disease		qualitative binding	Positive	
420681	2095961	H398, I399, Q520, T521, T537, N556, K558	Occurrence of infectious disease	73, 74, 195, 196, 212, 231, 233	qualitative binding	Positive	
420685	2095967	Y394, F409, V438, G439, T441, D442	Occurrence of infectious disease	69, 84, 113, 114, 116, 117	qualitative binding	Positive	
434425	2480854	D385, R393, G423, H495, M496, K558, K559	Occurrence of infectious disease	60, 68, 98, 170, 171, 233, 234	neutralization	Positive	26159721
	2480855				qualitative binding	Positive	
	2480856				neutralization	Positive	
	2480857				neutralization	Positive	
	2480858				neutralization	Positive	
	2480859				neutralization	Positive	
434426	2480860	D388, W389, T390, R405, I446, A487, N518	Occurrence of infectious disease	63, 64, 65, 80, 121, 162, 193	qualitative binding	Positive	26159721
	2480861				neutralization	Positive	
	2480862				qualitative binding	Positive	
	2480863				dissociation constant KD	Positive	
	2480864				neutralization	Positive	
	2480865				neutralization	Positive	
	2480866				neutralization	Positive	
	2480867				neutralization	Positive	
	2482652				on rate	Positive	

	2482653				off rate	Positive	
434427	2480881	D499, R523, Y524, K540	Occurrence of infectious disease	174, 198, 199, 215	neutralization	Positive	26159721
	2480882				qualitative binding	Positive	
	2480883				neutralization	Positive	
	2480884				neutralization	Positive	
	2480885				neutralization	Positive	
434428	2469561	D575	Occurrence of infectious disease	250	survival after pathogen challenge after adoptive transfer	Positive	26159721
	2480812				neutralization	Positive	
	2480813				qualitative binding	Positive	
	2480814				dissociation constant KD	Positive	
	2480815				neutralization	Positive	
	2480816				neutralization	Positive	
	2480817				neutralization	Positive	
	2480818				neutralization	Positive	
	2482650				on rate	Positive	
	2482651				off rate	Positive	
434430	2469560	G578	Occurrence of infectious disease	253	survival after pathogen challenge after adoptive transfer	Positive	26159721
	2480779				qualitative binding	Positive	
	2480780				neutralization	Positive	
	2480781				qualitative binding	Positive	
	2480782				dissociation constant KD	Positive	
	2480783				qualitative binding	Positive-Low	
	2480786				dissociation constant KD	Positive	

	2480790				neutralization	Positive	
	2480791				neutralization	Positive	
	2480792				neutralization	Positive	
	2480793				neutralization	Positive	
	2482642				on rate	Positive	
	2482643				off rate	Positive	
	2482644				on rate	Positive	
	2482645				off rate	Positive	
434431	2480800	H343	Occurrence of infectious disease	18	qualitative binding	Positive	26159721
	2480801				neutralization	Positive	
	2480802				qualitative binding	Positive	
	2480803				dissociation constant KD	Positive	
	2482692				on rate	Positive	
	2482693				off rate	Positive	
434432	2480834	H387, W389, D442	Occurrence of infectious disease	62, 64, 117	qualitative binding	Positive	26159721
	2480835				neutralization	Positive	
	2480836				qualitative binding	Positive	
	2480837				dissociation constant KD	Positive	
	2480838				neutralization	Positive	
	2480839				neutralization	Positive	
	2480840				neutralization	Positive	
	2480841				neutralization	Positive	
	2482654				on rate	Positive	
	2482655				off rate	Positive	
434433	2480826	H387, W389, R393, H424, D442, I580	Occurrence of infectious disease	62, 64, 68, 99, 117, 255	qualitative binding	Positive	26159721
	2480827				neutralization	Positive	
	2480828				qualitative binding	Positive	
	2480829				dissociation constant KD	Positive	

	2480830				neutralization	Positive	
	2480831				neutralization	Positive	
	2480832				neutralization	Positive	
	2480833				neutralization	Positive	
	2482700				on rate	Positive	
	2482701				off rate	Positive	
434434	2480794	I515, R523, Y524, G534, L535, T537	Occurrence of infectious disease	190, 198, 199, 209, 210, 212	qualitative binding	Positive	26159721
	2480795				neutralization	Positive	
	2480796				qualitative binding	Positive	
	2480797				dissociation constant KD	Positive	
	2480798				neutralization	Positive	
	2480799				neutralization	Positive	
	2482694				on rate	Positive	
	2482695				off rate	Positive	
434435	2480848	M496, Q509, I515, N518, V522, R523, Y524, G534, L535, K540, K559, V567, I580	Occurrence of infectious disease	171, 184, 190, 193, 197, 198, 199, 209, 210, 215, 234, 242, 255	qualitative binding	Positive	26159721
	2480849				neutralization	Positive	
	2480850				qualitative binding	Positive	
	2480851				neutralization	Positive	
	2480852				neutralization	Positive	
	2480853				neutralization	Positive	
434436	2481097	R361, H387, R405, Q471, E490, E491, N556, D575, H581	Occurrence of infectious disease	36, 62, 80, 146, 165, 166, 231, 250, 256	neutralization	Positive	26159721
	2481098				qualitative binding	Positive	
	2481099				dissociation constant KD	Positive	
	2482696				on rate	Positive	
	2482697				off rate	Positive	
434437	2481088	R405	Occurrence of infectious disease	80	neutralization	Positive-Low	26159721
	2481089				qualitative binding	Positive	
	2481094				neutralization	Positive	

	2481095				neutralization	Positive	
434438	2480804	R405, T441	Occurrence of infectious disease	80, 116	qualitative binding	Positive	26159721
	2480805				neutralization	Positive	
	2480806				qualitative binding	Positive	
	2480808				neutralization	Positive	
	2480809				neutralization	Positive	
	2480810				neutralization	Positive	
	2480811				neutralization	Positive	
434439	2480819	T383, D384, D385, R393, D396, I399, D402, T516, N518, K559	Occurrence of infectious disease	58, 59, 60, 68, 71, 74, 77, 191, 193, 234	qualitative binding	Positive	26159721
	2480820				neutralization	Positive	
	2480821				qualitative binding	Positive	
	2480822				neutralization	Positive	
	2480823				neutralization	Positive	
	2480824				neutralization	Positive	
	2480825				neutralization	Positive	
434440	2469562	W389	Occurrence of infectious disease	64	survival after pathogen challenge after adoptive transfer	Positive	26159721
	2480868				neutralization	Positive	
	2480869				qualitative binding	Positive	
	2480870				dissociation constant KD	Positive	
	2480871				neutralization	Positive	
	2480872				neutralization	Positive	
	2480873				neutralization	Positive	
	2480874				neutralization	Positive	
	2482646				on rate	Positive	
	2482647				off rate	Positive	
434441	2480842	W389, D396, R405, T441, D442, I446, N512,	Occurrence of infectious	64, 71, 80, 116, 117, 121, 187,	neutralization	Positive	26159721
	2480843				qualitative	Positive	

		I515	disease	190	binding		
	2480844				neutralization	Positive	
	2480845				neutralization	Positive	
	2480846				neutralization	Positive	
	2480847				neutralization	Positive	
434442	2481086	W389, T390	Occurrence of infectious disease	64,65	neutralization	Positive	26159721
	2481087				qualitative binding	Positive	
	2481090				neutralization	Positive	
	2481091				neutralization	Positive	
	2481092				neutralization	Positive	
462266	2546041	N7, V8, K10, A11, D59, D60, H62, E166, V169, M171, G194, T212, H232, K234, W235, R251, K252, G253, K254, I255	Occurrence of infectious disease	7, 8, 10, 11, 59, 60, 62, 166, 169, 171, 194, 212, 232, 234, 235, 251, 252, 253, 254, 255	3D structure	Positive	26504196
	2546048		Occurrence of infectious disease		qualitative binding	Positive	
	2546057		Occurrence of infectious disease		neutralization	Positive	
462267	2546043	N7, V8, K10, A11, R13, K57, T58, D59, D60, S61, D63, K66, H73, M74, P75, A76, D77, V169, M171, N193, G194, Q195, V229, N231, H232, K233, K234, W235, G253	Occurrence of infectious disease	7, 8, 10, 11, 13, 57, 58, 59, 60, 61, 63, 66, 73, 74, 75, 76, 77, 169, 171, 193, 194, 195, 229, 231, 232, 233, 234, 235, 253	3D structure	Positive	26504196
	2546049		Occurrence of infectious disease		qualitative binding	Positive	26504196
	2546058		Occurrence of infectious disease		neutralization	Positive	26504196
462515	2546065	F6, N7, V8, Y9, K10, T12, T58, D59, D60, S61, H62, N193, G194, Q195, H232, K234	Occurrence of infectious disease	6, 7, 8, 9, 10, 12, 58, 59, 60, 61, 62, 193, 194, 195, 232, 234	3D structure	Positive	26537684

462516	2546064	K200, G204, G205, S206, E208, K215, V216, I217, N218, N219	Occurrence of infectious disease	200, 204, 205, 206, 208, 215, 216, 217, 218, 219	3D structure	Positive	26537684
956298	6353122	K233	Occurrence of infectious disease	233	neutralization	Positive	31697791
	6353126				dissociation constant KD	Positive	
	6353128				neutralization	Positive	
	6353134				survival after pathogen challenge after adoptive transfer	Positive	
	6353136				qualitative binding	Positive	
956299	6353123	N231, K233	Occurrence of infectious disease	231, 233	neutralization	Positive	31697791
	6353127				dissociation constant KD	Positive	
	6353129				neutralization	Positive	
	6353135				survival after pathogen challenge after adoptive transfer	Positive-Low	
