# Supplementary material for: Predicting Immunogenic Epitopes Variation of Envelope 2 Gene Among Chikungunya Virus Clonal Lineages by an In Silico Approach
Source: Viruses. 2024 Oct 29;16(11):1689. doi: 10.3390/v16111689 (PMC11599094; doi:10.3390/v16111689)
Supplement: Supplementary file 1 [file viruses-16-01689-s001.zip › Table S3.pdf]

Table S3. MHC class I binding epitopes E2 of East Central South African (ECSA) strain (GenBank no. NP 690589) predicted by the NetMHCPan 4 at IEDB (http://www.iedb.org).

HLA allele	Start	End	Length	Epitope peptide[a]	IC50	Percentile rank
HLA-A*11:01	1	10	10	STKDNFNVYK	6.76	0.02
HLA-A*30:01	1	10	10	STKDNFNVYK	8.9	0.03
HLA-A*68:01	1	10	10	STKDNFNVYK	13.69	0.13
HLA-A*31:01	1	10	10	STKDNFNVYK	25.89	0.18
HLA-A*30:02	1	9	9	STKDNFNVY	46.98	0.06
HLA-A*03:01	1	10	10	STKDNFNVYK	47.62	0.15
HLA-A*33:01	4	13	10	DNFNVYKATR	29.39	0.05
HLA-A*33:01	5	13	9	NFNVYKATR	13.16	0.02
HLA-B*15:01	6	15	10	FNVYKATRPY	25.12	0.09
HLA-B*35:01	7	15	9	NVYKATRPY	49.46	0.1
HLA-A*02:06	48	56	9	IQVSLQIGI	38.6	0.36
HLA-A*68:01	60	68	9	DSHDWTKLR	46.07	0.45
HLA-A*33:01	60	68	9	DSHDWTKLR	46.29	0.09
HLA-A*02:03	69	78	10	YMDNHIPADA	35.94	0.6
HLA-B*07:02	74	83	10	IPADAGRAGL	14.01	0.05
HLA-A*31:01	78	86	9	AGRAGLFVR	25.84	0.18
HLA-A*68:02	94	103	10	TGTMGHFILA	33.84	0.2
HLA-A*31:01	95	104	10	GTMGHFILAR	11.13	0.05
HLA-A*11:01	95	104	10	GTMGHFILAR	12.23	0.05
HLA-A*02:06	95	103	9	GTMGHFILA	13.01	0.12
HLA-A*68:01	95	104	10	GTMGHFILAR	19.91	0.2
HLA-A*02:03	95	103	9	GTMGHFILA	44	0.73
HLA-A*30:01	95	103	9	GTMGHFILA	48.04	0.25
HLA-A*31:01	96	104	9	TMGHFILAR	29.96	0.22

HLA-A*68:01	110	119	10	TLTVGFTDSR	6.99	0.05
HLA-A*68:01	111	119	9	LTVGFTDSR	6.29	0.04
HLA-A*68:01	111	120	10	LTVGFTDSRK	29.36	0.32
HLA-B*15:01	121	129	9	ISHSCTHPF	28.96	0.09
HLA-B*35:01	127	135	9	HPFHHDPPV	38.92	0.09
HLA-A*02:03	180	188	9	LLSQQSGNV	23.24	0.39
HLA-A*68:01	190	198	9	ITVNSQTVR	41.91	0.42
HLA-A*11:01	191	200	10	TVNSQTVRYK	11.62	0.04
HLA-A*68:01	191	200	10	TVNSQTVRYK	23.88	0.25
HLA-A*03:01	191	200	10	TVNSQTVRYK	47.79	0.15
HLA-B*58:01	226	235	10	HAAVTNHKKW	19.36	0.1
HLA-A*68:01	226	234	9	HAAVTNHKK	27.14	0.3
HLA-A*31:01	235	244	10	WQYNSPLVPR	38.37	0.27
HLA-A*33:01	235	244	10	WQYNSPLVPR	44.43	0.08
HLA-A*31:01	236	244	9	QYNSPLVPR	24.39	0.17
HLA-A*33:01	236	244	9	QYNSPLVPR	38.8	0.07
HLA-B*07:02	239	248	10	SPLVPRNAEL	8.32	0.03
HLA-A*30:01	254	262	9	KIHIPFPLA	10.4	0.03
HLA-A*68:02	256	264	9	HIPFPLANV	14.05	0.1
HLA-A*02:03	256	264	9	HIPFPLANV	37.91	0.63
HLA-A*02:03	259	268	10	FPLANVTCMV	3.1	0.03
HLA-B*35:01	259	267	9	FPLANVTCM	4.81	0.02
HLA-A*02:01	259	268	10	FPLANVTCMV	6.14	0.05
HLA-A*02:06	259	268	10	FPLANVTCMV	15.26	0.14
HLA-B*53:01	259	267	9	FPLANVTCM	31.2	0.05
HLA-B*07:02	268	276	9	VPKARNPTV	45.37	0.12
HLA-A*30:01	270	278	9	KARNPTVTY	21.37	0.09
HLA-A*30:01	270	279	10	KARNPTVTYG	38.47	0.19
HLA-A*30:02	280	288	9	KNQVIMLLY	46.35	0.06

HLA-A*02:01	285	294	10	MLLYPDHPTL	47.04	0.41
HLA-A*02:03	286	295	10	LLYPDHPTLL	9.25	0.12
HLA-A*02:01	286	294	9	LLYPDHPTL	17.46	0.16
HLA-A*02:03	286	294	9	LLYPDHPTL	21.23	0.36
HLA-A*02:06	286	294	9	LLYPDHPTL	29.49	0.26
HLA-A*02:01	286	295	10	LLYPDHPTLL	36.72	0.33
HLA-B*35:01	288	297	10	YPDHPTLLSY	7.06	0.02
HLA-B*53:01	288	297	10	YPDHPTLLSY	41.61	0.05
HLA-A*30:02	298	306	9	RSMGEEPNY	42.61	0.06
HLA-B*53:01	321	330	10	VPTEGLEVTW	18.87	0.03
HLA-B*58:01	321	330	10	VPTEGLEVTW	41.83	0.17
HLA-A*11:01	328	337	10	VTWGNNEPYK	26.39	0.13

a; Predicted epitopes were filtered by the percentile rank <1 and IC50 ≤50nM.
