# Supplementary material for: Predicting Immunogenic Epitopes Variation of Envelope 2 Gene Among Chikungunya Virus Clonal Lineages by an In Silico Approach
Source: Viruses. 2024 Oct 29;16(11):1689. doi: 10.3390/v16111689 (PMC11599094; doi:10.3390/v16111689)
Supplement: Supplementary file 1 [file viruses-16-01689-s001.zip › Table S5.pdf]

Table S5. MHC class I binding epitopes E2 of Asian strain (GenBank no. ACY66830) predicted by the NetMHCPan 4 at IEDB (http://www.iedb.org).

HLA allele	Start	End	Length	Epitope peptide[a]	IC50	Percentile rank
HLA-A*11:01	1	10	10	SIKDHFNVYK	9.88	0.03
HLA-A*30:01	1	10	10	SIKDHFNVYK	19.4	0.08
HLA-A*03:01	1	10	10	SIKDHFNVYK	25.1	0.06
HLA-A*68:01	1	10	10	SIKDHFNVYK	29.9	0.32
HLA-B*15:01	1	9	9	SIKDHFNVY	32	0.11
HLA-A*31:01	1	10	10	SIKDHFNVYK	35.3	0.25
HLA-A*33:01	4	13	10	DHFNVYKATR	30.3	0.05
HLA-A*33:01	5	13	9	HFNVYKATR	15.6	0.03
HLA-A*31:01	5	13	9	HFNVYKATR	18.5	0.12
HLA-B*15:01	6	15	10	FNVYKATRPY	25.1	0.09
HLA-B*35:01	7	15	9	NVYKATRPY	49.5	0.1
HLA-A*02:06	48	56	9	IQVSLQIGI	38.6	0.36
HLA-A*68:01	60	68	9	DSHDWTKLR	46.1	0.45
HLA-A*33:01	60	68	9	DSHDWTKLR	46.3	0.09
HLA-A*30:01	68	76	9	RYMDNHMPA	48.1	0.25
HLA-B*07:02	74	83	10	MPADAERAGL	15.6	0.05
HLA-A*68:02	94	103	10	TGTMGHFILA	33.8	0.2
HLA-A*31:01	95	104	10	GTMGHFILAR	11.1	0.05
HLA-A*11:01	95	104	10	GTMGHFILAR	12.2	0.05
HLA-A*02:06	95	103	9	GTMGHFILA	13	0.12
HLA-A*68:01	95	104	10	GTMGHFILAR	19.9	0.2
HLA-A*02:03	95	103	9	GTMGHFILA	44	0.73
HLA-A*30:01	95	103	9	GTMGHFILA	48	0.25
HLA-A*31:01	96	104	9	TMGHFILAR	30	0.22
HLA-A*68:01	110	119	10	TLTVGFTDGR	7.12	0.05
HLA-A*68:01	111	119	9	LTVGFTDGR	5.79	0.03
HLA-A*68:01	111	120	10	LTVGFTDGRK	40.9	0.41
HLA-B*15:01	121	129	9	ISHSCTHPF	29	0.09
HLA-B*35:01	127	135	9	HPFHHDPPV	38.9	0.09
HLA-A*31:01	140	149	10	KFHSRPQHGR	33.5	0.24
HLA-A*02:03	179	188	10	TLMSQQSGNV	18.9	0.31
HLA-A*02:03	180	188	9	LMSQQSGNV	24.9	0.42
HLA-A*68:01	190	198	9	ITVNSQTVR	41.9	0.42
HLA-A*11:01	191	200	10	TVNSQTVRYK	11.6	0.04

HLA-A*68:01	191	200	10	TVNSQTVRYK	23.9	0.25
HLA-A*03:01	191	200	10	TVNSQTVRYK	47.8	0.15
HLA-B*58:01	226	235	10	HAAVTNHKKW	19.4	0.1
HLA-A*68:01	226	234	9	HAAVTNHKK	27.1	0.3
HLA-A*31:01	235	244	10	WQYNSPLVPR	38.4	0.27
HLA-A*33:01	235	244	10	WQYNSPLVPR	44.4	0.08
HLA-A*31:01	236	244	9	QYNSPLVPR	24.4	0.17
HLA-A*33:01	236	244	9	QYNSPLVPR	38.8	0.07
HLA-B*07:02	239	248	10	SPLVPRNAEF	22.4	0.07
HLA-A*30:01	254	262	9	KVHIPFPLA	6.58	0.02
HLA-A*68:02	256	264	9	HIPFPLANV	14.1	0.1
HLA-A*02:03	256	264	9	HIPFPLANV	37.9	0.63
HLA-A*02:03	259	268	10	FPLANVTCRV	3.24	0.04
HLA-A*02:01	259	268	10	FPLANVTCRV	8.39	0.08
HLA-A*02:06	259	268	10	FPLANVTCRV	25.1	0.23
HLA-A*30:01	267	276	10	RVPKARNPTV	22.7	0.1
HLA-B*07:02	268	276	9	VPKARNPTV	45.4	0.12
HLA-A*30:01	270	278	9	KARNPTVTY	21.4	0.09
HLA-A*30:01	270	279	10	KARNPTVTYG	38.5	0.19
HLA-A*30:02	280	288	9	KNQVIMLLY	46.4	0.06
HLA-A*02:01	285	294	10	MLLYPDHPTL	47	0.41
HLA-A*02:03	286	295	10	LLYPDHPTLL	9.25	0.12
HLA-A*02:01	286	294	9	LLYPDHPTL	17.5	0.16
HLA-A*02:03	286	294	9	LLYPDHPTL	21.2	0.36
HLA-A*02:06	286	294	9	LLYPDHPTL	29.5	0.26
HLA-A*02:01	286	295	10	LLYPDHPTLL	36.7	0.33
HLA-B*35:01	288	297	10	YPDHPTLLSY	7.06	0.02
HLA-B*53:01	288	297	10	YPDHPTLLSY	41.6	0.05
HLA-B*53:01	321	330	10	VPTEGLEVTW	18.9	0.03
HLA-B*58:01	321	330	10	VPTEGLEVTW	41.8	0.17
HLA-A*11:01	328	337	10	VTWGNNEPYK	26.4	0.13

a; Predicted epitopes were filtered by the percentile rank <1 and IC50 ≤50nM.
