# Supplementary material for: Predicting Immunogenic Epitopes Variation of Envelope 2 Gene Among Chikungunya Virus Clonal Lineages by an In Silico Approach
Source: Viruses. 2024 Oct 29;16(11):1689. doi: 10.3390/v16111689 (PMC11599094; doi:10.3390/v16111689)
Supplement: Supplementary file 1 [file viruses-16-01689-s001.zip › Table S6.pdf]

Table S6. List of T cell epitopes of Chikungunya virus mature E2 protein identified from IEDB assay database (http://www.iedb.org)

IEDB Epitope ID	IEDB Assay ID	Predicted MHC hot spot in this study	Name	Starting Position of E2	Ending Position of E2	Process Type	Method	Response measured
169782	22441610	MHC I & II hot spot 1	STKDNFNVYK	1	10	Prophylactic vaccination	biological activity	activation
2191694	22406615	MHC I & II hot spot 1	STKDNFNVYKATRPY	1	15	Occurrence of infectious disease	ELISPOT	IFNg release
	22441613					Prophylactic vaccination	biological activity	activation
2252980	22441358	MHC I & II hot spot 1	FNVYKATRPYLAHCP	6	20	Prophylactic vaccination	biological activity	activation
2253056	22441523	MHC I & II hot spot 1	NVYKATRPY	7	15	Prophylactic vaccination	biological activity	activation
2189548	22441291		ATDGTLKIQVSLQIG	41	55	Prophylactic vaccination	biological activity	activation
2191578	22406579	MHC I hot spot 2	SHDWTKLRYMDNHMP	61	75	Occurrence of infectious disease	ELISPOT	IFNg release
2190586	22441449	MHC I hot spot 2	KLRYMDNHMPADAER	66	80	Prophylactic vaccination	biological activity	activation
2189424	22405823	Partial MHC I hot spot 2	ADAERAGLFVRTSAP	76	90	Occurrence of infectious disease	ELISPOT	IFNg release
2191491	22441581	MHC I hot spot 2 & 3	RTSAPCTITGTMGHF	86	100	Prophylactic vaccination	biological activity	activation
2189667	22441313	MHC I hot spot 2 & 3	CTITGTMGHFILARC	91	105	Prophylactic vaccination	biological activity	activation
2191815	22406661	Partial MHC I hot spot 3	TMGHFILARCPKGET	96	110	Occurrence of infectious disease	ELISPOT	IFNg release
	22441630					Prophylactic vaccination	biological activity	activation
2190887	22700838	MHC I hot spot 4	LTVGFTDSRKISHSC	111	125	Vaccination	ELISPOT	IFNg release
	22700839					Vaccination	ELISPOT	IFNg release
2191783	22406646	Partial MHC I hot spot 4	THPFHHDPPVIGREK	126	140	Occurrence of infectious disease	ELISPOT	IFNg release
	22700840					Vaccination	ELISPOT	IFNg release
	22700841					Vaccination	ELISPOT	IFNg release
2252997	22441398	MHC I hot spot 4	HPFHHDPPV	127	135	Prophylactic vaccination	biological activity	activation
2190294	22406130	Partial MHC I hot spot 4	HDPPVIGREKFHSRP	131	145	Occurrence of infectious disease	ELISPOT	IFNg release
2190966	22406351	MHC I hot spot 5	MSQQSGNVKITVNSQ	181	195	Occurrence of infectious disease	ELISPOT	IFNg release
2190238	22406114	MHC I hot spot 5	GNVKITVNSQTVRYK	186	200	Occurrence of infectious disease	ELISPOT	IFNg release

2190468	22406191		ITTDKVINNCKID QC	211	225	Occurrence of infectious disease	ELISPOT	IFNg release
2191869	22406683		TTTDKVINNCKVD QC	211	225	Occurrence of infectious disease	ELISPOT	IFNg release
2190563	22406221	Partial MHC I hot spot 6	KIDQCHAAVTNH KKW	221	235	Occurrence of infectious disease	ELISPOT	IFNg release
2190640	22406247	Partial MHC I hot spot 6	KVDQCHAAVTNH KKW	221	235	Occurrence of infectious disease	ELISPOT	IFNg release
2190289	22406127	MHC I hot spot 6	HAAVTNHKKWQ YNSP	226	240	Occurrence of infectious disease	ELISPOT	IFNg release
2252994	22441395	MHC I hot spot 7	HIPFPLANV	256	264	Prophylactic vaccination	biological activity	activation
2190673	22441470	MHC I hot spot 7	LANVTCRVPKAR NPT	261	275	Prophylactic vaccination	biological activity	activation
2253121	22441675	MHC I hot spot 7	VPKARNPTV	268	276	Prophylactic vaccination	biological activity	activation
	22441676					Prophylactic vaccination	biological activity	activation
2189525	22441281	MHC I hot spot 7	ARNPTVTYGKNQ VIM	271	285	Prophylactic vaccination	biological activity	activation
2192092	22441691	MHC I hot spot 7	VTYGKNQVIMLL YPD	276	290	Prophylactic vaccination	biological activity	activation
2191085	22441518	MHC I hot spot 7	NQVIMLLYPDHPT LL	281	295	Prophylactic vaccination	biological activity	activation
2253044	22441499	MHC I hot spot 7	MLLYPDHPTL	285	294	Prophylactic vaccination	biological activity	activation
2190783	22441483	MHC II hot spot 2	LLYPDHPTLLSYR NM	286	300	Prophylactic vaccination	biological activity	activation
	22700842					Vaccination	ELISPOT	IFNg release
	22700843					Vaccination	ELISPOT	IFNg release
2190320	22407158	Partial MHC II hot spot 2	HPTLLSYRNMGEE PN	291	305	Occurrence of infectious disease	ELISPOT	IFNg release
	22441405					Prophylactic vaccination	biological activity	activation
2191724	22441616	Partial MHC II hot spot 2	SYRNMGEEPNYQ EEW	296	310	Prophylactic vaccination	biological activity	activation
2192078	22406742	Partial MHC I hot spot 8	VTHKKEIRLTVPT EG	311	325	Occurrence of infectious disease	ELISPOT	IFNg release
2189909	22405987	Partial MHC I hot spot 8	EIRLTVPTEGLEVT W	316	330	Occurrence of infectious disease	ELISPOT	IFNg release
2192046	22441678	MHC I hot spot 8	VPTEGLEVTWGN NEP	321	335	Prophylactic vaccination	biological activity	activation
2252977	22441341	Partial MHC I hot spot 8	EPYKYWPQL	334	342	Prophylactic vaccination	biological activity	activation
